# Supplementary figures and images for: Generation of mitochondrial reactive oxygen species is controlled by ATPase inhibitory factor 1 and regulates cognition
Source: PLoS Biol. 2021 May 13;19(5):e3001252. doi: 10.1371/journal.pbio.3001252 (PMC8148373; doi:10.1371/journal.pbio.3001252)

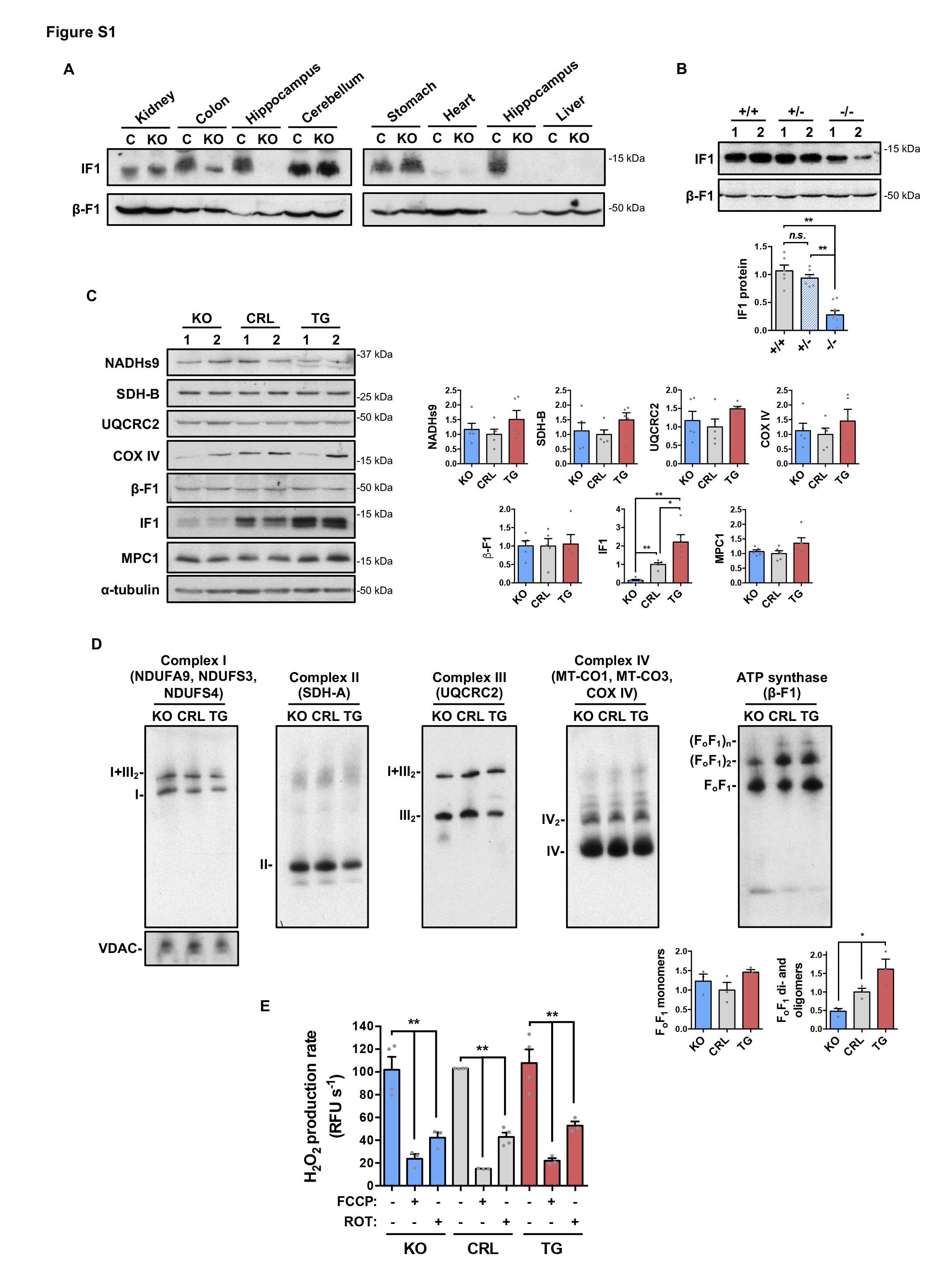

Supplement: S1 Fig — (A) Representative western blot of IF1 and β-F1 expression in different brain regions and tissues from CRL (C) and IF1KO mice. (B) Representative western blot of IF1 in forebrain extracts from CRL (+/+), heterozygous (+/−) and IF1KO (−/−) mice. Two independent samples are shown (1 and 2). The histogram to the left shows the quantification of IF1/β-F1 ratio (n = 6–8). There is some IF1 expression in IF1KO mice because the Cre recombinase is expressed in excitatory neurons, hence other neuronal types have not undergone recombination. (C) Western blot analysis of the expression of OXPHOS complexes I (NADHs9), II (SDH-B), III (UQCRC2), IV (COX IV), ATP synthase (β-F1), IF1, and MPC1 in hippocampal extracts of IF1KO, CRL, and IF1TG mice. α-tubulin is shown as control, and 2 representative samples are shown (1 and 2). Histograms to the right show the quantification as fold change of CRL (n = 5). (D) Representative BN immunoblots probed with antibodies against mitochondrial complexes I (NDUFA9, NDUFS3 and NDUFS4), II (SDH-A), III (UQCRC2), IV (COX IV, MT-CO1 and MT-CO3), and ATP synthase (β-F1). The migration of oligomers (FoF1)n, dimers (FoF1)2 and monomers (FoF1) of the ATP synthase and other superassemblies is indicated. VDAC is shown as control. Histograms to the bottom show the quantification of ATP synthase monomers and superassemblies (dimers and oligomers) as fold change of CRL (n = 3). (E) The histograms show the rate of H2O2 production in isolated forebrain mitochondria using succinate as respiratory substrate in the absence or presence of FCCP or rotenone (n = 3–4). Error bars: mean ± SEM. *P < 0.05, **P < 0.01 by 2-tailed t test (B–E). Related to Fig 2. Uncropped western blots can be found in S1 Raw Images, and numerical data underlying plots in S1 Data. BN, blue native; CRL, control; IF1, ATPase inhibitory factor 1; IF1KO, IF1 knockout; IF1TG, IF1 overexpressing transgenic; MPC1, mitochondrial pyruvate carrier 1; OXPHOS, oxidative phosphorylation; VDAC, voltag [file pbio.3001252.s001.tif]

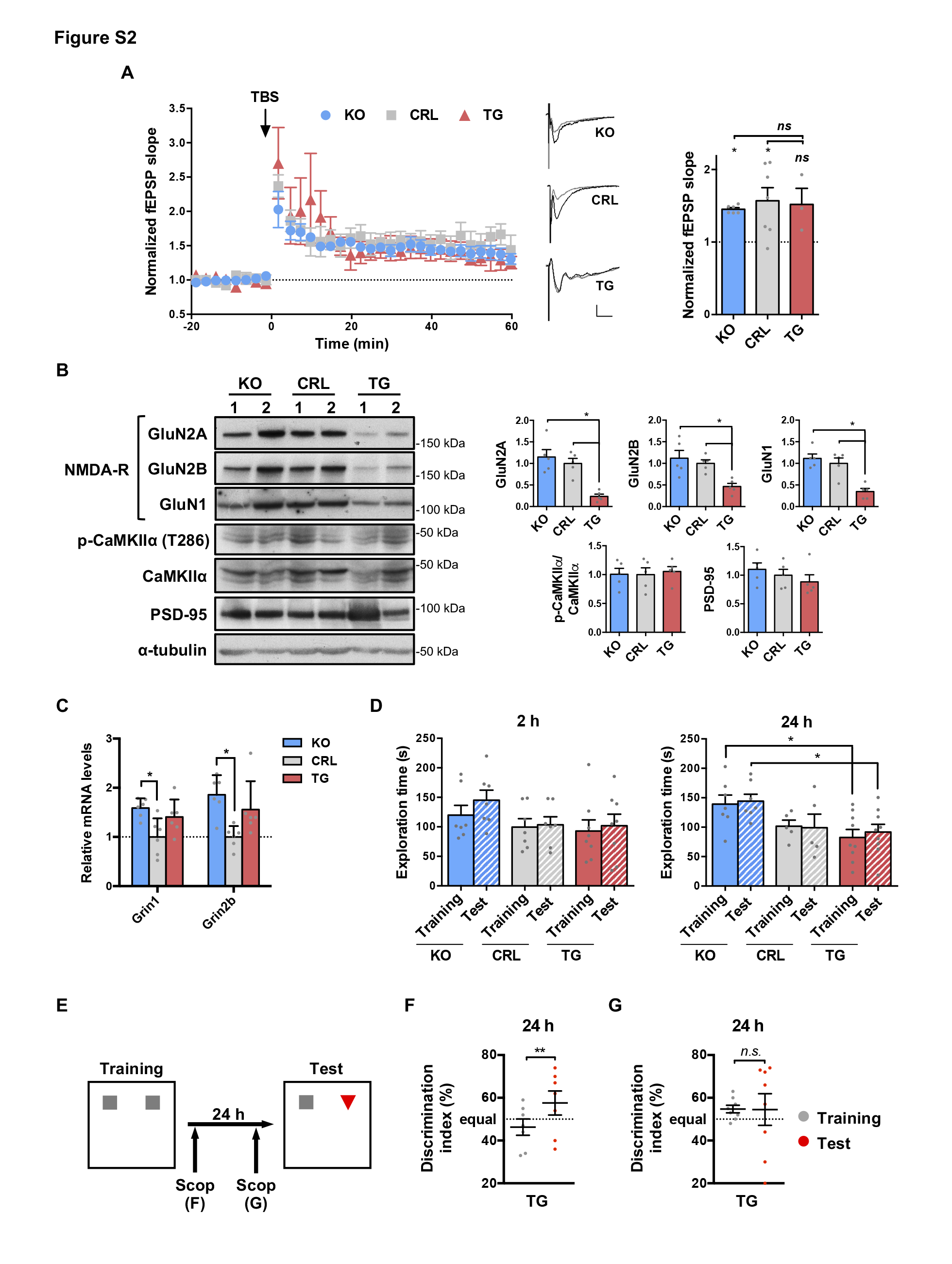

Supplement: S2 Fig — (A) Time course of the relative changes in fEPSP slope before and after TBS in hippocampal slices from IF1KO and CRL (n = 6–7 slices from 4 mice) and IF1TG mice (n = 3 slices from 3 mice). Insets: representative fEPSPs before (gray line) and after (black) TBS. The histogram to the right summarizes the last 5 minutes of the time courses shown in (A). (B) Western blots of the phosphorylation and expression of NMDA receptor subunits 2A, 2B, and 1 (GluN2A, GluN2B, and GluN1), calcium/Camk2a and PSD-95. α-tubulin is shown as control, and 2 representative samples are shown (1 and 2). Histograms to the right show the quantification as fold of CRL (n = 5). (C) qPCR analysis of the mRNA levels of NMDA receptor subunits 1 (Grin1) and 2B (Grin2b) in IF1KO, CRL, and IF1TG mice (n = 6). (D) Total time spent exploring both objects in the training or test sessions of the short- (left) and long-term (right) memory tests for IF1KO, CRL (n = 7 each), and IF1TG mice (n = 8). (E) Long-term memory tests with Scop administration. (F and G) Box plots show the discrimination indexes when Scop was injected after the training (F) or before the test session (G) to IF1TG mice (n = 8). Error bars: mean ± SEM. *P < 0.05, **P < 0.01 by 2-tailed (–-D) or pairwise (F and G) t tests, or Kruskal–Wallis followed by Dunn multiple comparisons test (A); Wilcoxon test was used to analyze LTP expression with respect to baseline (A). Related to Fig 6. Uncropped western blots can be found in S1 Raw Images, and numerical data underlying plots in S1 Data. Camk2a, Calcium/calmodulin-dependent protein kinase II α; CRL, control; fEPSP, field excitatory postsynaptic potential; IF1, ATPase inhibitory factor 1; IF1KO, IF1 knockout; IF1TG, IF1 overexpressing transgenic; PSD-95, postsynaptic density protein 95; qPCR, quantitative polymerase chain reaction; Scop, scopolamine; TBS, theta burst stimulation. (TIF) [file pbio.3001252.s002.tif]

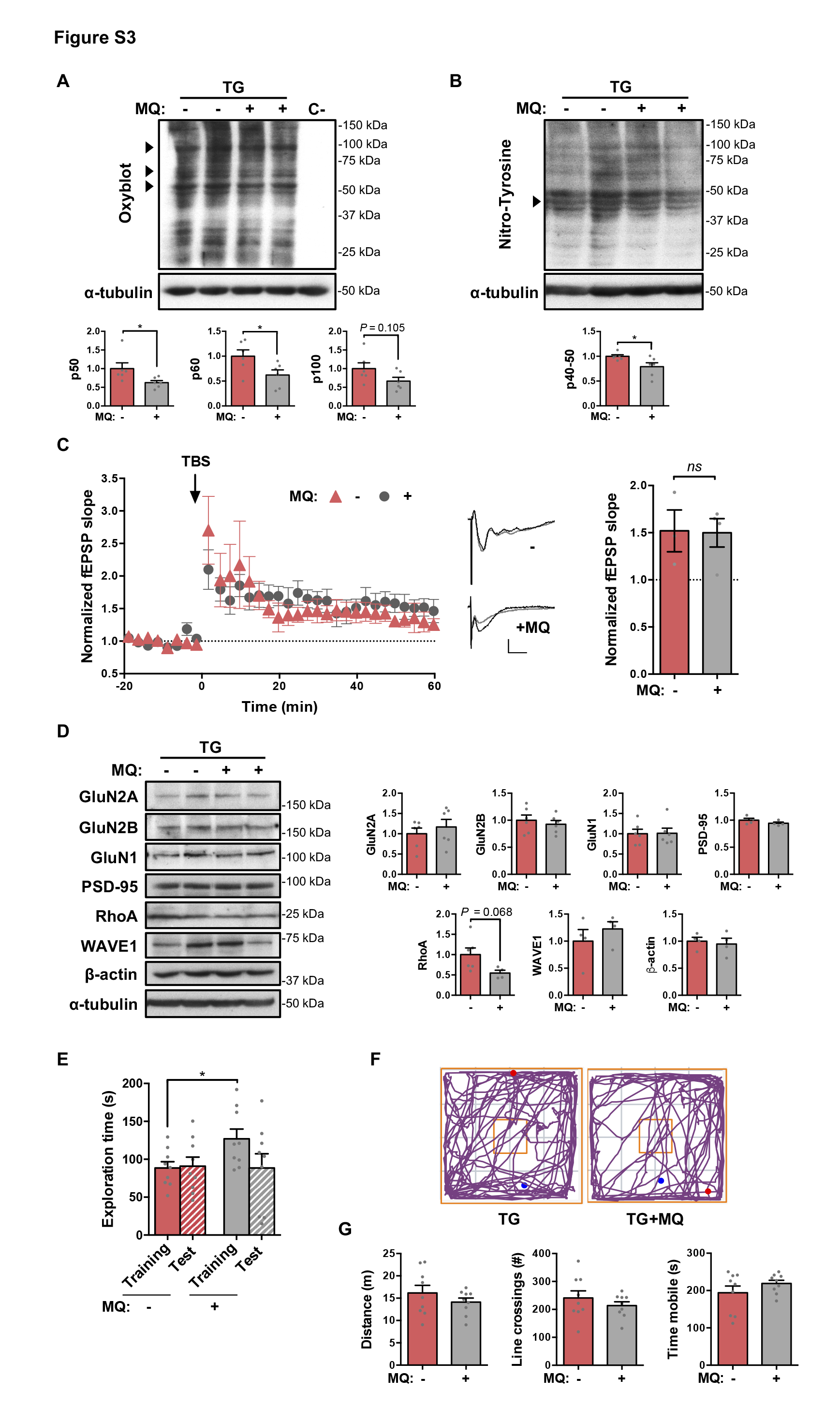

Supplement: S3 Fig — (A and B) Representative blots of the extent of carbonylation (A) and tyrosine nitration (B) of hippocampal proteins. α-tubulin is shown as control. Arrowheads to the left of the blots identify the migration of the proteins used in the quantification of carbonylation and nitration (histograms to the bottom, n = 6). (C) Time course of relative changes in fEPSP slope before and after TBS in hippocampal slices from IF1TG mice untreated (n = 3 slices from 3 mice) or treated with MQ (n = 4 slices from 4 mice). Insets: representative fEPSPs before (gray line) and after (black) TBS. The histogram to the right summarizes the results from the last 5 minutes of the time courses. (D) Western blots of glutamate receptor NMDA subunits 2A, 2B, and 1 (GluN2A, GluN2B, and GluN1), PSD-95, WAVE1, RhoA, and β-actin. α-tubulin is shown as control. Histograms to the right show the quantification as fold of untreated IF1TG mice (n = 4–6). (E) Total time spent exploring both objects in the training and test sessions of the long-term memory test (n = 9). (F) Representative track plots showing the position of the center of IF1TG mice during the open field test. Blue and red dots show the start and end points of the track, respectively. (G) No significant differences were found in distance traveled, number of lines of the virtual grid crossed, or time mobile (n = 9). Error bars: mean ± SEM. *P < 0.05 by 2-tailed t test (A, B, D, and E) or 2-way ANOVA with Bonferroni multiple comparisons test (C). In (C), data from IF1TG mice are replotted from S2A Fig, since the recordings were performed in the same experiment. Related to Fig 7. Uncropped western blots can be found in S1 Raw Images, and numerical data underlying plots in S1 Data. fEPSP, field excitatory postsynaptic potential; IF1TG, IF1 overexpressing transgenic; MQ, MitoQ; PSD-95, postsynaptic density protein 95; RhoA, Ras homolog family member A; TBS, theta burst stimulation; WAVE1, WASP family member 1. (TIF) [file pbio.3001252.s003.tif]

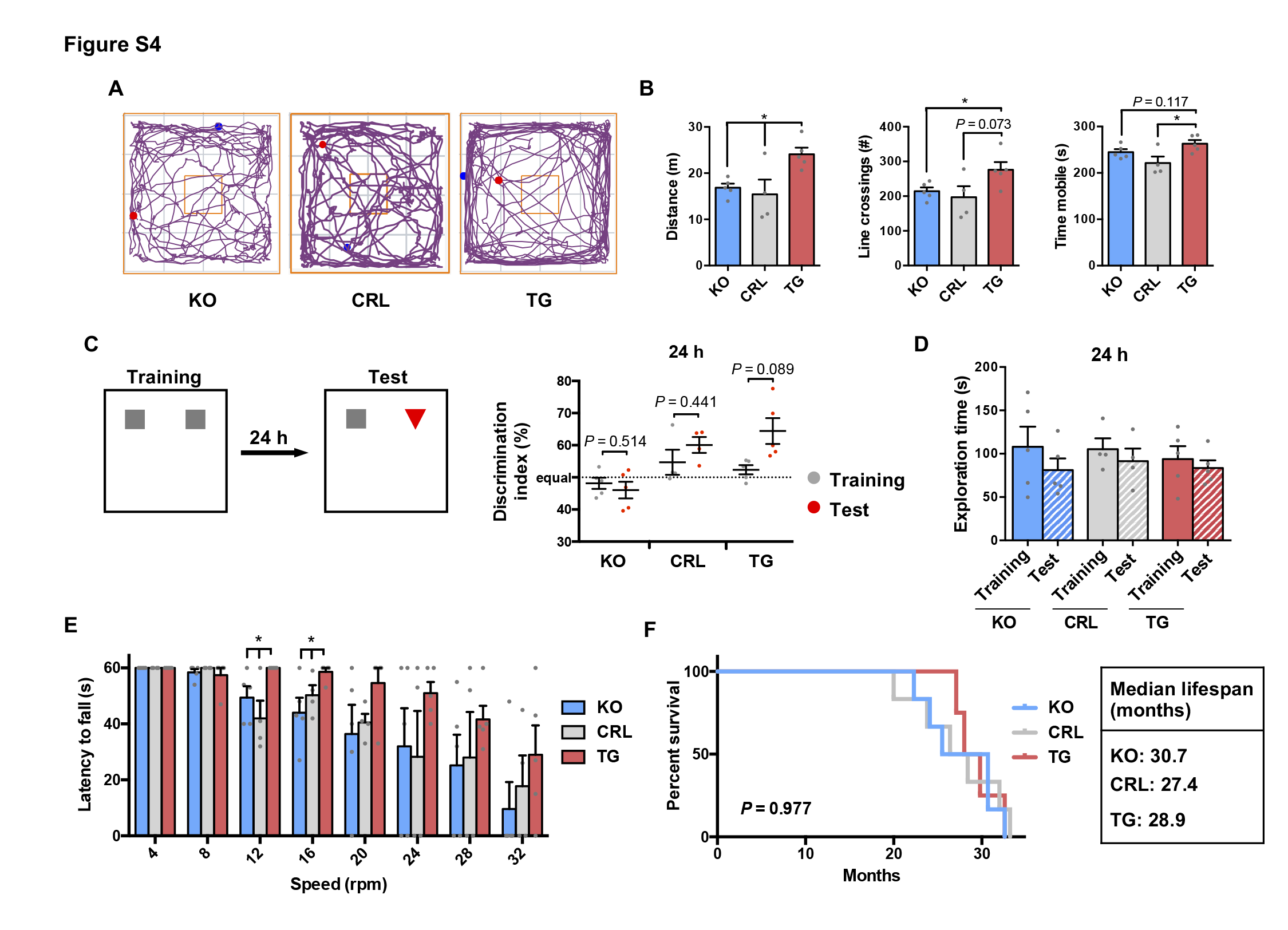

Supplement: S4 Fig — (A) Representative track plots showing the position of the center of approximately 2-year-old IF1KO, CRL, and IF1TG mice in the open field test. Blue and red dots show the start and end points of the track, respectively. (B) Histograms show total distance traveled, number of lines of the virtual grid crossed, and time mobile. (C) Schematic of the long-term memory test. The plot to the right shows the discrimination index, calculated as the relative time spent exploring the novel object with respect to the total time exploring both objects. (D) No significant differences were found in total time exploring both objects. (E) The histogram shows latency to fall in the Rota-rod test. (F) Survival analysis by Kaplan–Meier and log-rank tests of IF1KO (n = 6), CRL (n = 6), and IF1TG mice (n = 4). The median life span of each genotype is shown to the right. Error bars: mean ± SEM for IF1KO (n = 5), CRL (n = 4), and IF1TG mice (n = 5). *P < 0.05 by 2-tailed (B, E, and D) or pairwise (C) t tests. Numerical data underlying plots can be found in S1 Data. CRL, control; IF1, ATPase inhibitory factor 1; IF1KO, IF1 knockout; IF1TG, IF1 overexpressing transgenic. (TIF) [file pbio.3001252.s004.tif]

Figure 1

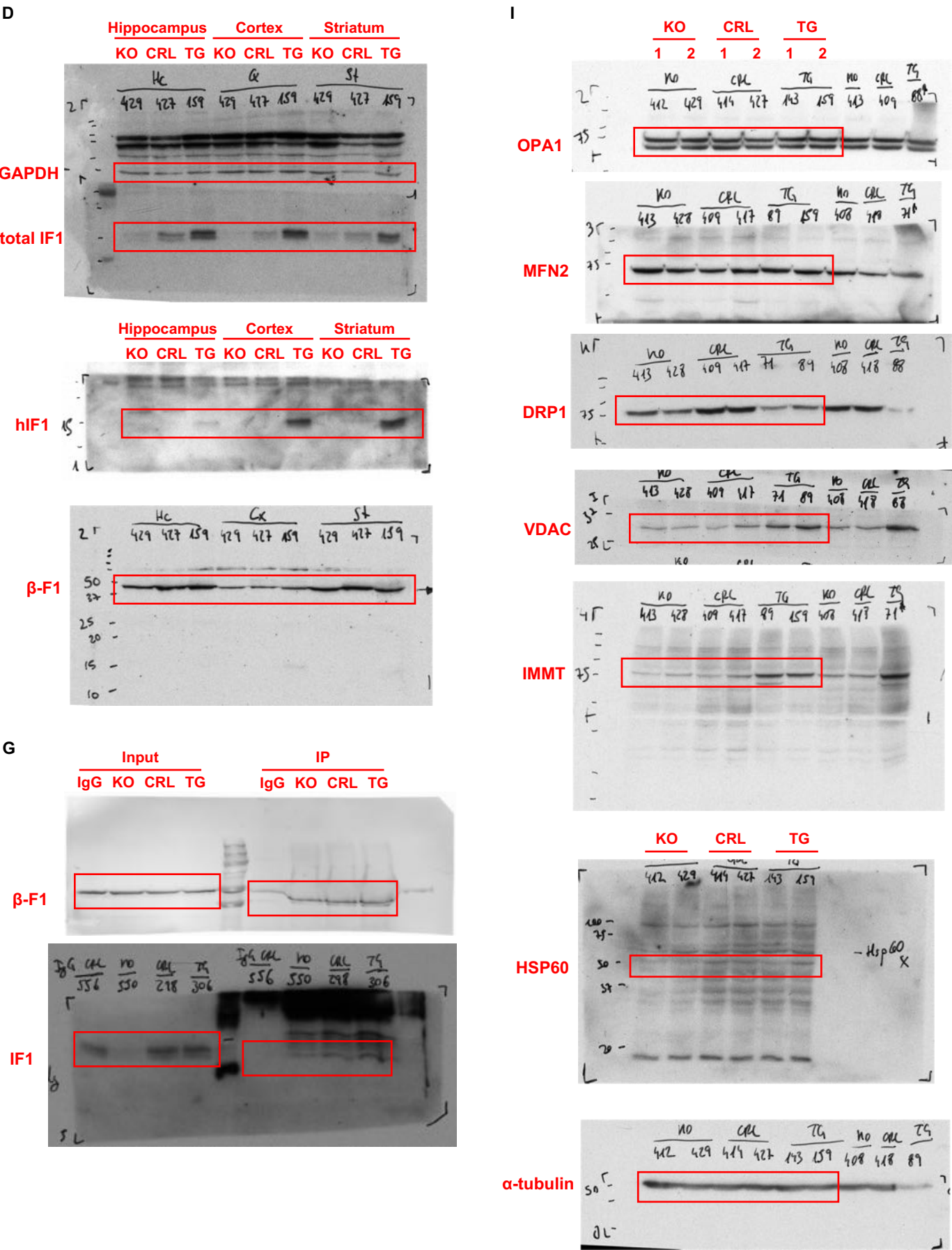

Figure 2

G

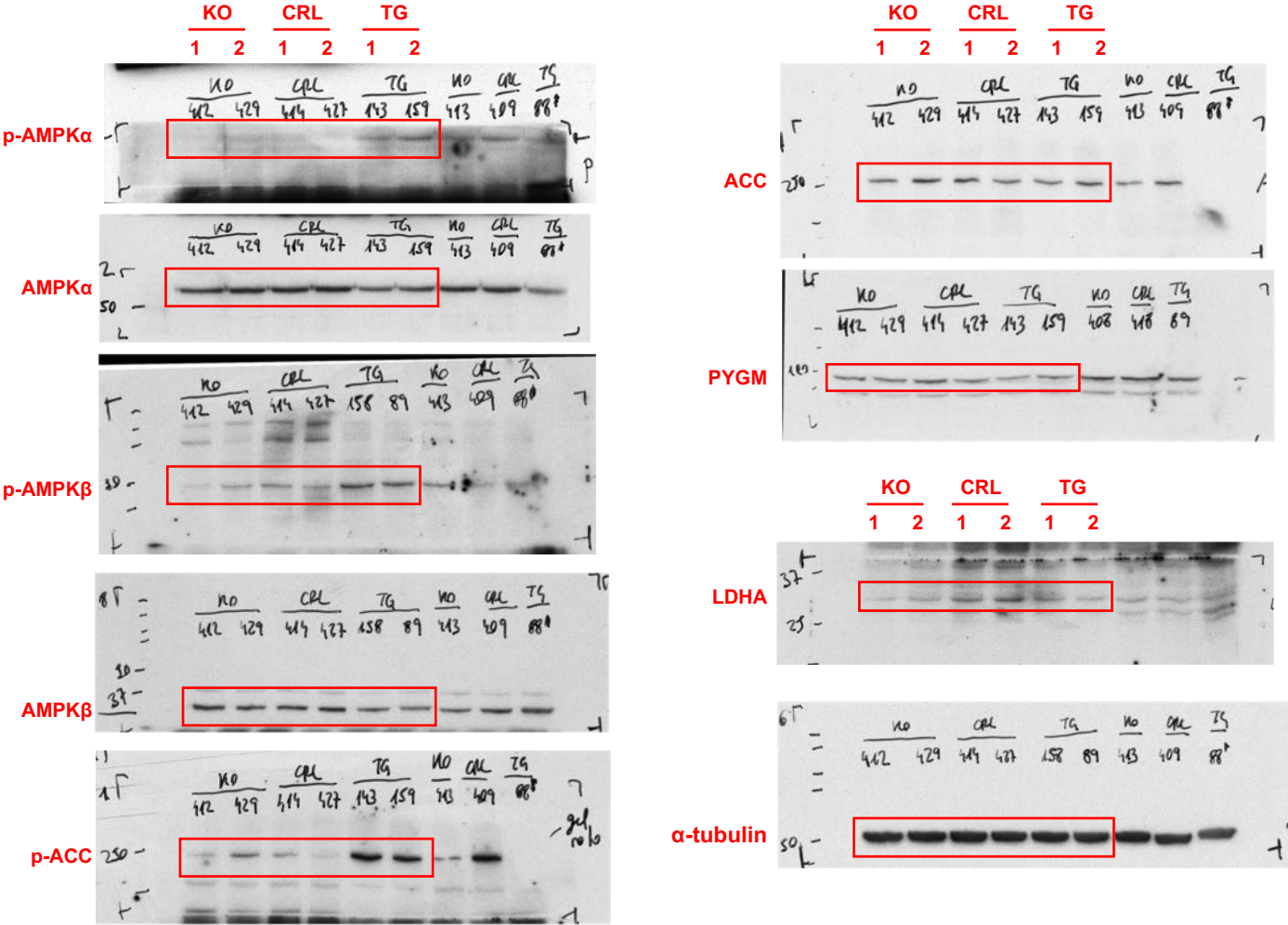

Figure 2

J

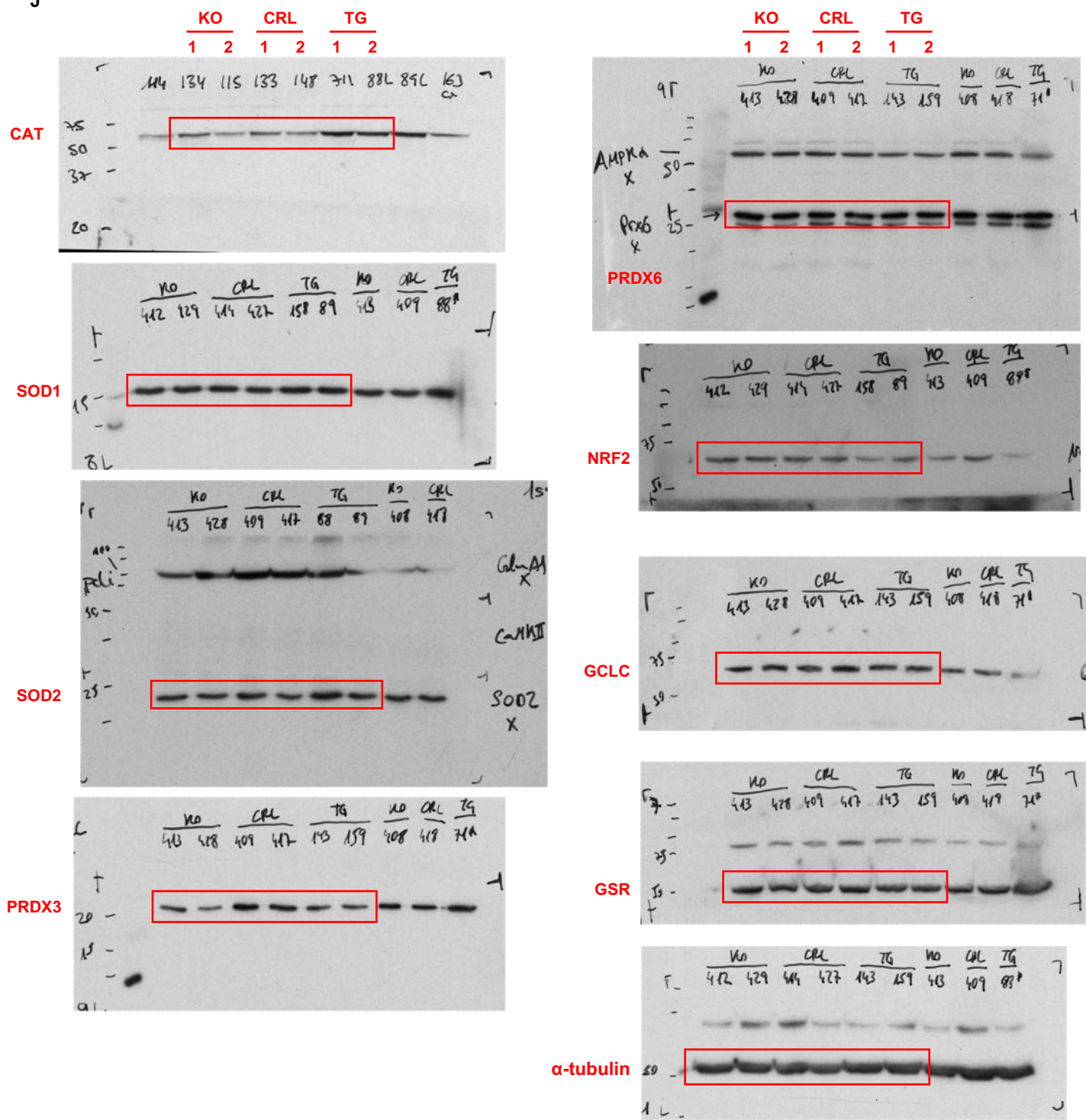

Figure 6

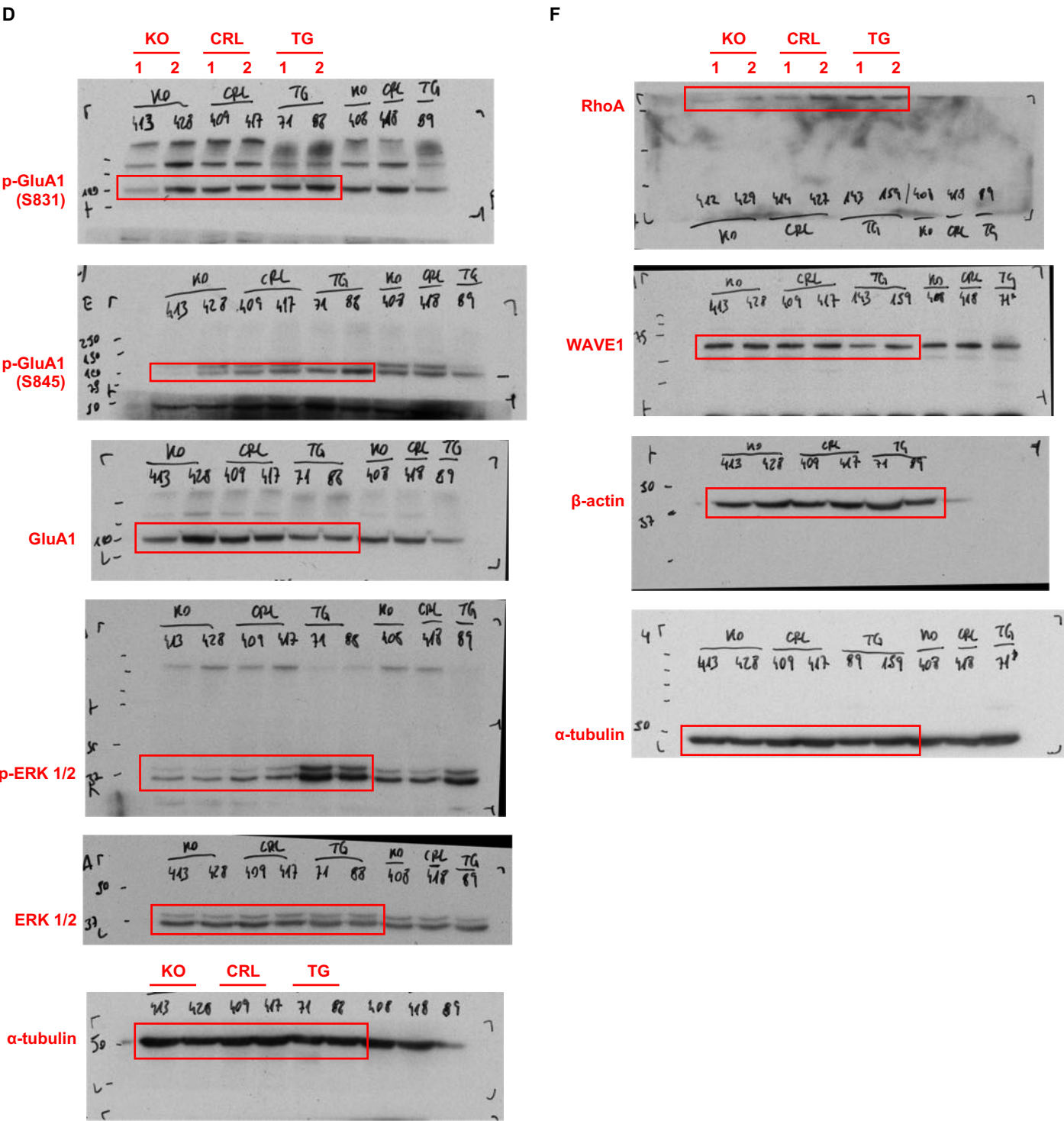

Figure 7

B

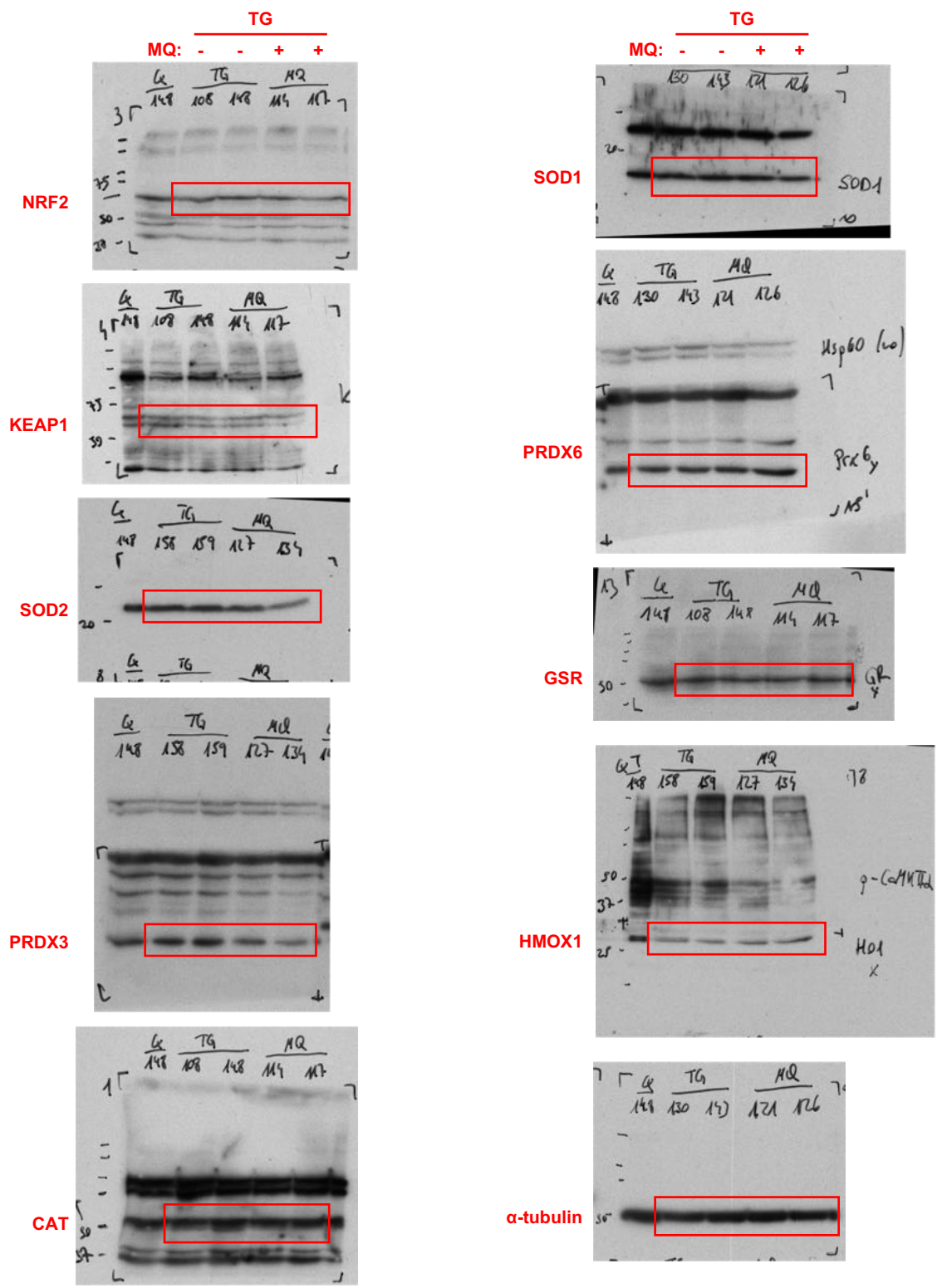

Figure 7

D

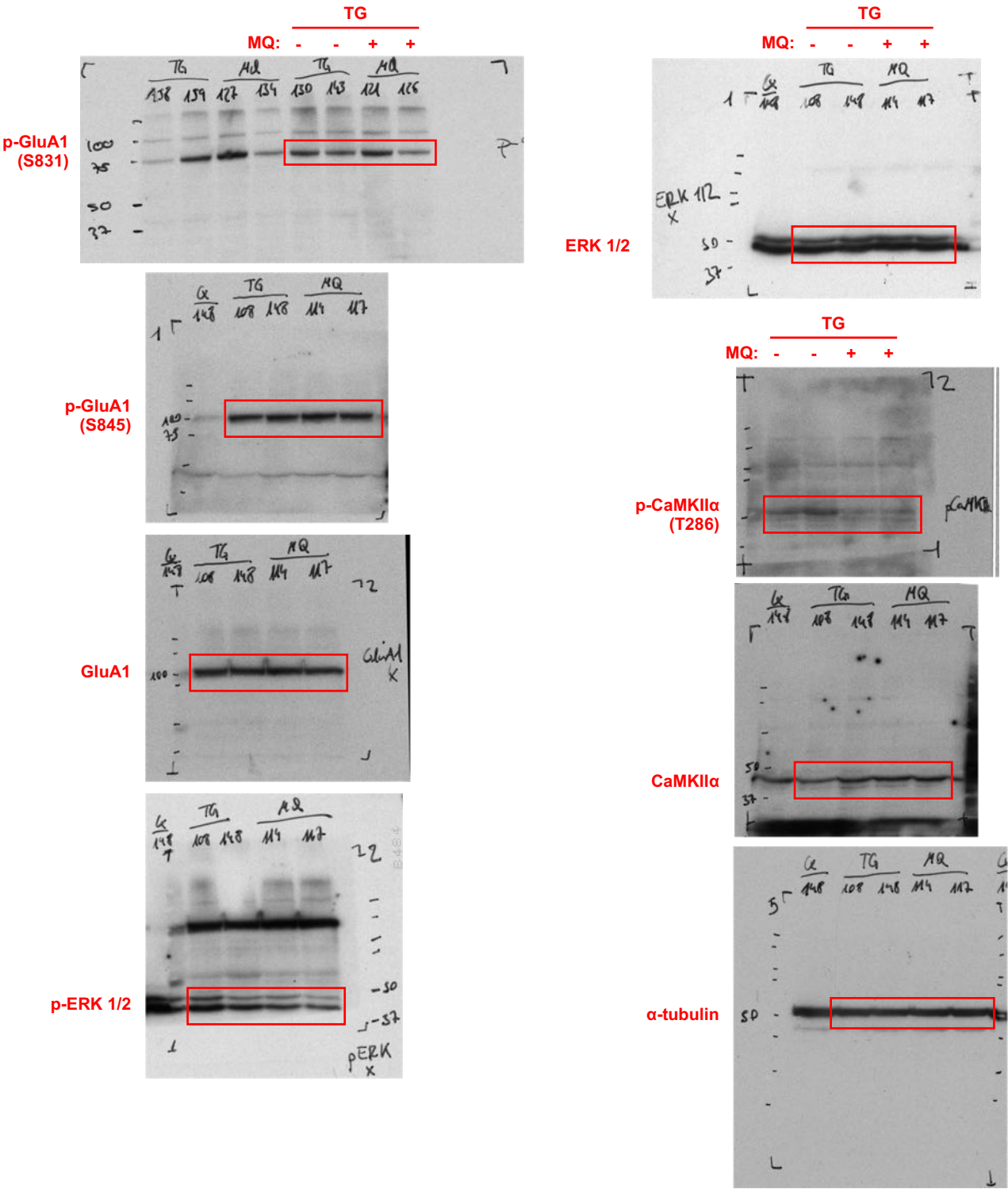

Figure S1

A

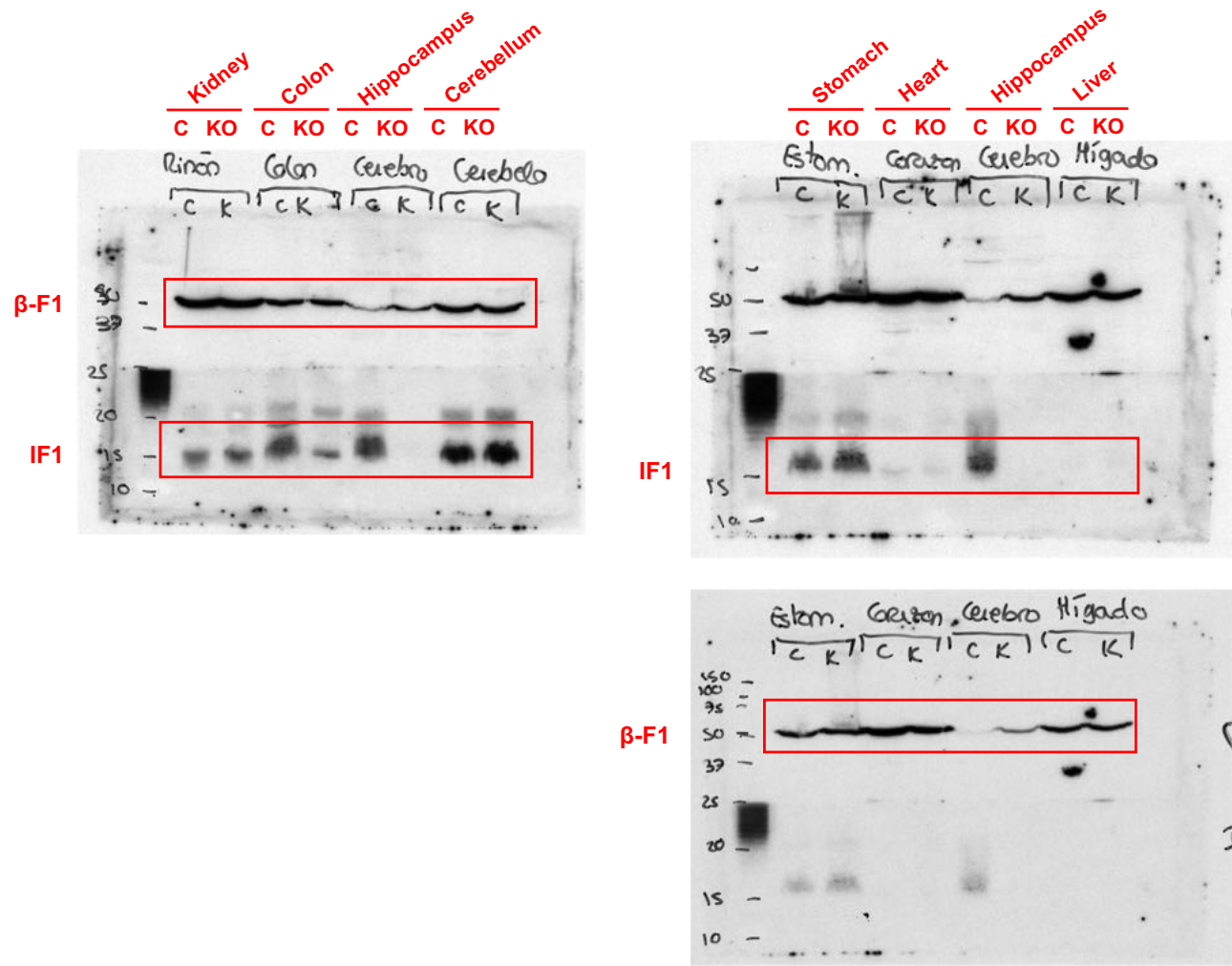

B

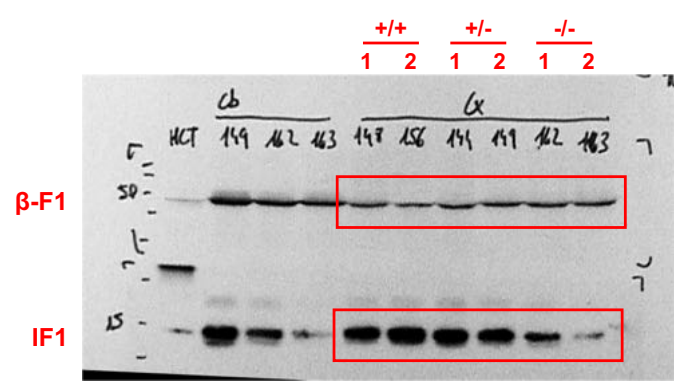

Figure S1

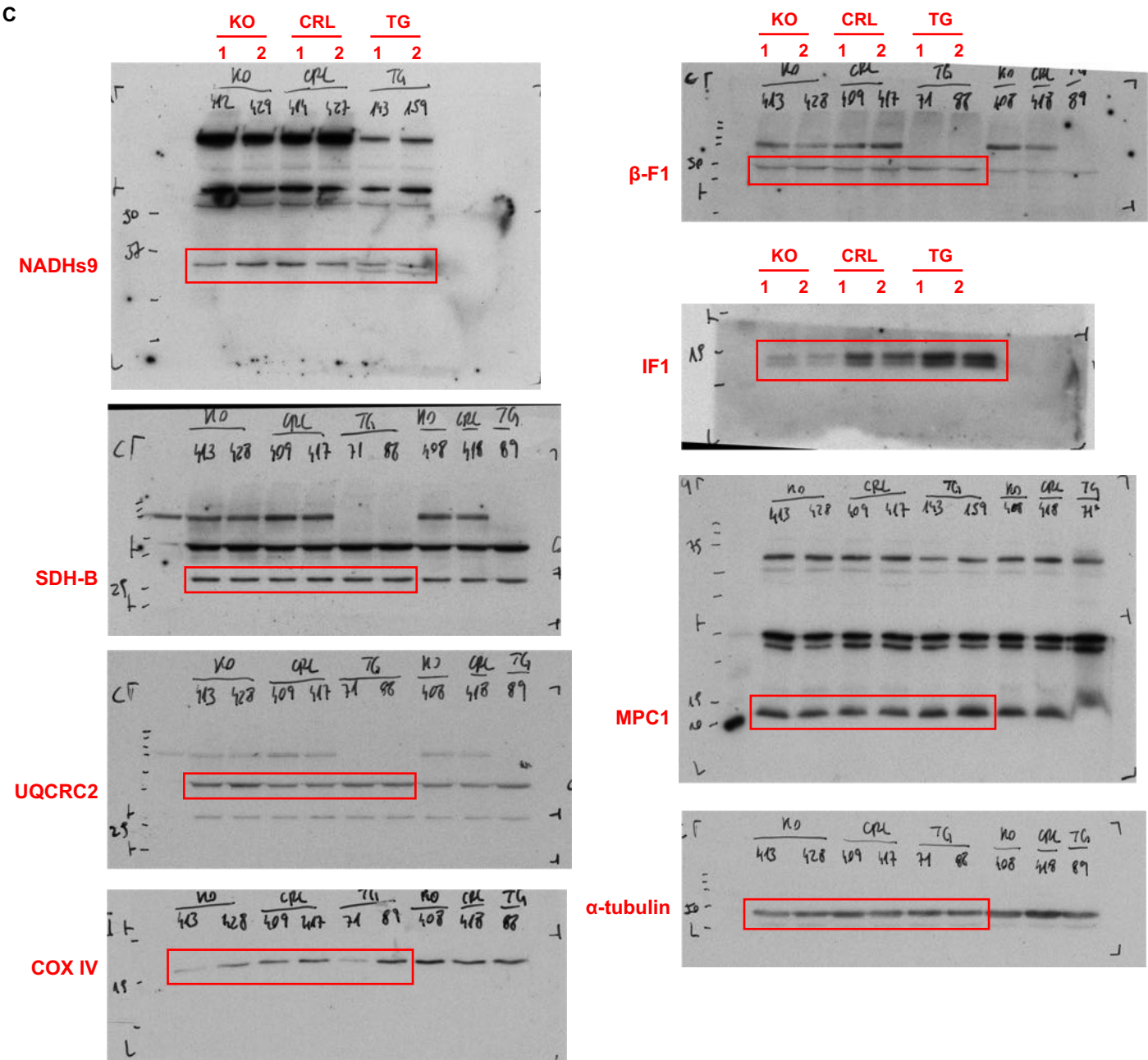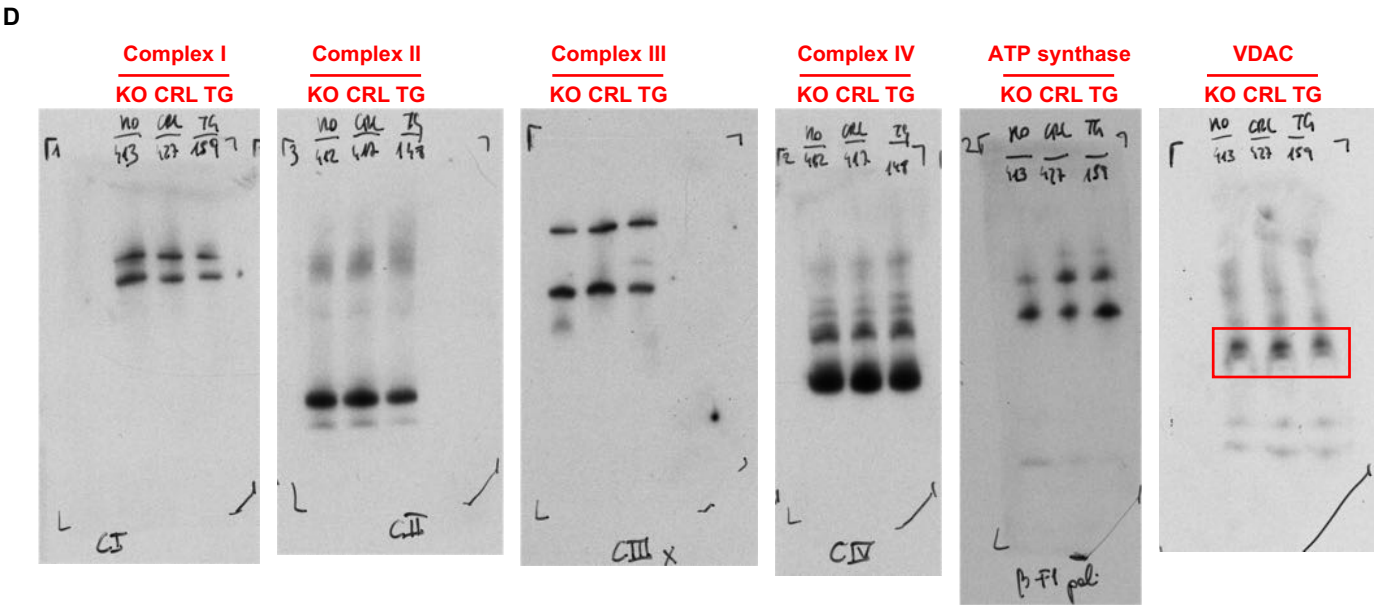

Figure S2

B

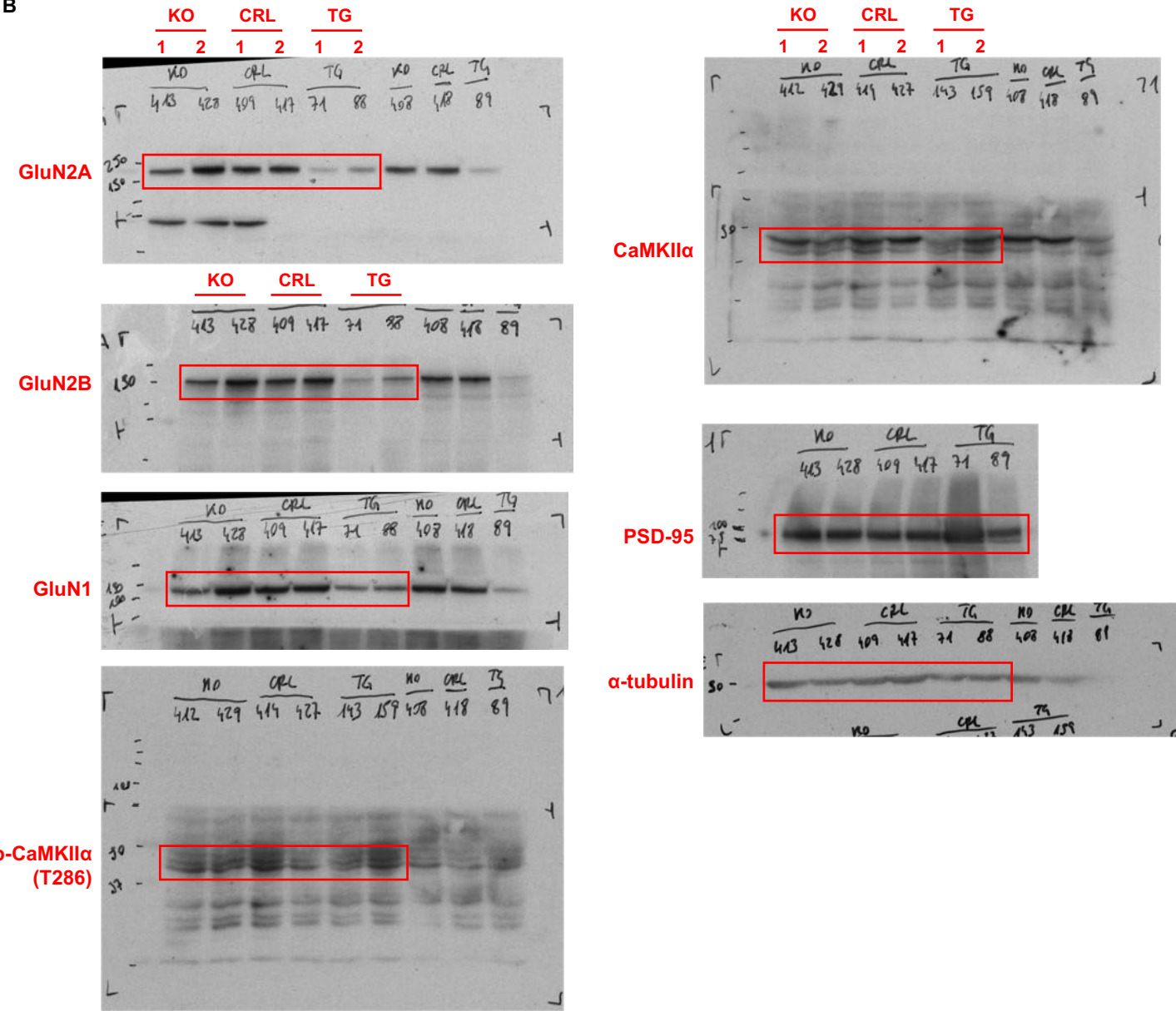

Figure S3

A

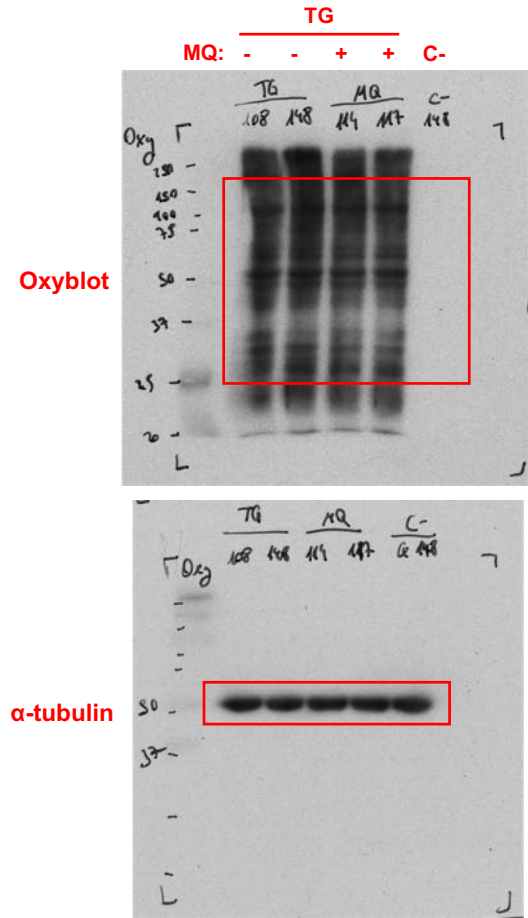

B

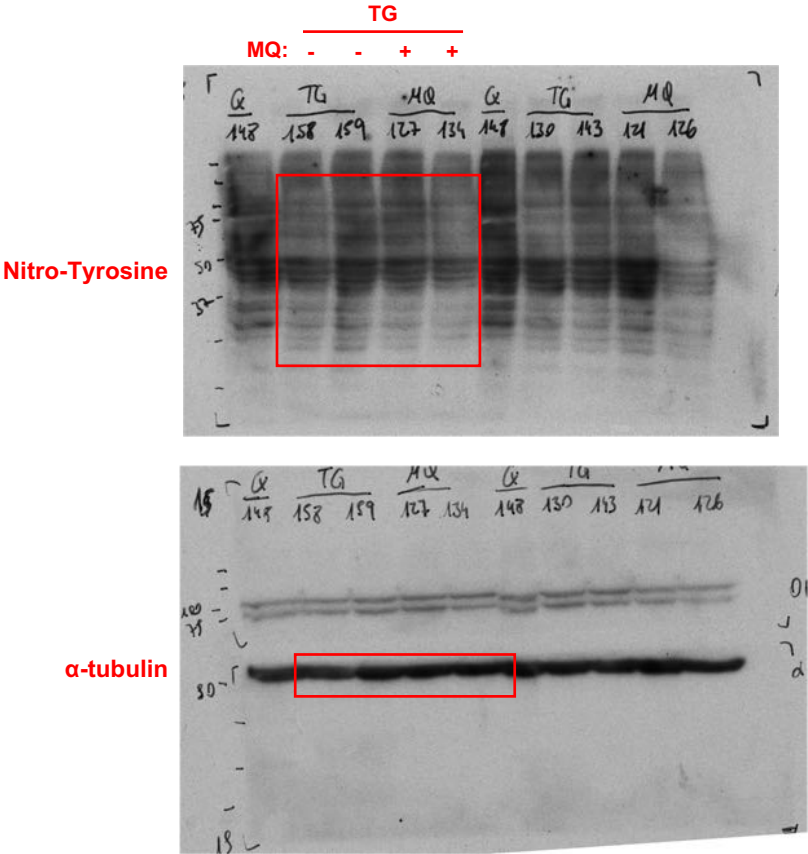

D

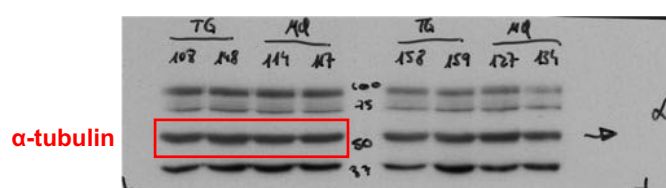

Supplement: S1 Raw Images — (PDF) [file pbio.3001252.s006.pdf]
