# Supplementary material for: Generation of mitochondrial reactive oxygen species is controlled by ATPase inhibitory factor 1 and regulates cognition
Source: PLoS Biol. 2021 May 13;19(5):e3001252. doi: 10.1371/journal.pbio.3001252 (PMC8148373; doi:10.1371/journal.pbio.3001252)
Supplement: S1 Table — The table summarizes the levels of other metabolites determined in hippocampal extracts. The results shown are the mean values ± SEM. Values are expressed as nmol per g, except for 3-hydroxyisobutyrate, 2-methyl-3-hydroxybutyrate, malate, and citrate, which are expressed as fold change of control. *P < 0.05 with respect to control, †P < 0.05 with respect to IF1KO by 2-tailed t test. IF1KO, IF1 knockout. (DOCX) [file pbio.3001252.s007.docx]

**S1 Table. Hippocampal metabolites.**

|  | ***IF1^KO^*** | **Control** | ***IF1^TG^*** |
| --- | --- | --- | --- |
| **Amino acids:** | | | |
| Valine | 168 ± 7 | 180 ± 3 | 152 ± 3 * |
| Leucine | 237 ± 8 | 248 ± 5 | 214 ± 3 * ^†^ |
| Isoleucine | 97 ± 2 | 101 ± 3 | 89 ± 2 * ^†^ |
| Tryptophan | 46 ± 1 | 51 ± 2 | 40 ± 3 * |
| Lysine | 333 ± 7 | 336 ± 12 | 319 ± 12 |
| Arginine | 315 ± 11 | 342 ± 44 | 322 ± 20 |
| Histidine | 89 ± 9 * | 139 ± 8 | 99 ± 2 * |
| Cysteine | 22 ± 3 | 25 ± 5 | 23 ± 3 |
| Methionine | 115 ± 6 | 123 ± 5 | 105 ± 4 * |
| Serine | 1,037 ± 4 * | 1,305 ± 79 | 989 ± 17 * |
| Threonine | 380 ± 13 * | 424 ± 5 | 357 ± 6 * |
| Proline | 130 ± 3 * | 161 ± 3 | 146 ± 6 |
| 4-Hydroxyproline | 2.6 ± 1.0 | 2.6 ± 0.4 | 2.9 ± 1.0 |
| Taurine | 12,448 ± 66 | 12,954 ± 1039 | 12,022 ± 441 |
| Citrulline | 34 ± 7 | 48 ± 4 | 39 ± 7 |
| Ornithine | 40 ± 7 * | 62 ± 3 | 44 ± 1 * |
| Cystathionine | 17 ± 2 | 19 ± 1 | 19 ± 2 |
| β-Alanine | 910 ± 48 | 976 ± 29 | 811 ± 24 * |
| Anserine | 19 ± 4 * | 34 ± 3 | 36 ± 4 ^†^ |
| Sarcosine | 158 ± 5 | 175 ± 11 | 146 ± 3 |
| Phosphoethanolamine | 1,196 ± 91 | 1,273 ± 113 | 1,171 ± 70 |
| α-Aminobutyric acid | 3.7 ± 0.4 | 2.6 ± 0.4 | 3.7 ± 1.3 |
| γ-Aminobutyric acid | 3,143 ± 127 | 3,294 ± 164 | 3,155 ± 146 |
| **Neurotransmitters:** | | | |
| Serotonin | 3.3 ± 0.1 | 3.2 ± 0.1 | 3.0 ± 0.2 |
| 5-Hydroxyindoleacetic acid | 1.8 ± 0.0 | 1.8 ± 0.0 | 1.7 ± 0.1 |
| **Organic acids:** | | | |
| Lactate | 983 ± 122 | 884 ± 57 | 1,283 ± 247 |
| Fumarate | 40 ± 7 | 44 ± 7 | 31 ± 3 |
| Glutarate | 8.6 ± 0.3 | 8.9 ± 1.0 | 9.2 ± 1.3 |
| 2-Hydroxyglutarate | 41 ± 3 | 36 ± 3 | 40 ± 4 |
| 3-Hydroxy-3-methylglutarate | 4.0 ± 0.2 | 3.9 ± 0.3 | 3.1 ± 1.5 |
| Glycerate | 15 ± 1 | 16 ± 3 | 18 ± 1 |
| Glycolate | 257 ± 27 | 181 ± 55 | 196 ± 62 |
| Ethylmalonate | 5.5 ± 0.4 | 5.2 ± 0.4 | 5.7 ± 0.5 |
| Adipate | 3.7 ± 0.2 | 4.1 ± 0.3 | 4.1 ± 0.4 |
| N-acetylaspartate | 51 ± 14 | 32 ± 7 | 75 ± 15 |
| Suberic acid | 2.8 ± 0.1 | 3.4 ± 0.2 | 3.6 ± 0.6 |
| 3-Hydroxyisobutyrate | 1.5 ± 0.2 | 1.0 ± 0.5 | 1.3 ± 0.2 |
| 2-Methyl-3-hydroxybutyrate | 1.0 ± 0.0 | 1.0 ± 0.2 | 0.7 ± 0.4 |
| Citrate | 0.7 ± 0.1 | 1.0 ± 0.2 | 0.6 ± 0.1 |
| Malate | 1.3 ± 0.1 | 1.0 ± 0.2 | 1.1 ± 0.1 |
