# Supplementary material for: Generation of mitochondrial reactive oxygen species is controlled by ATPase inhibitory factor 1 and regulates cognition
Source: PLoS Biol. 2021 May 13;19(5):e3001252. doi: 10.1371/journal.pbio.3001252 (PMC8148373; doi:10.1371/journal.pbio.3001252)
Supplement: Attachment — Submitted filename: Answers to Reviewers.docx [file pbio.3001252.s008.docx]

**ANSWERS TO REVIEWERS**

***Reviewer #1:***

*In their manuscript titled "Mitochondrial reactive oxygen species generated by the IF1-inhibited pool of ATP synthase regulate cognition," Esparza-Moltó et al explore the implications varying doses of the mitochondrial protein IF1 on mitochondrial oxidative phosphorylation, neuronal function, and learning in mice. Previous studies with full-body IF1 KO mice had yielded conflicting results regarding its function and the effect of its KO. In this study, the authors generated forebrain-specific IF1 KO and human-IF1-overexpressing mice. As expected, they observed decreased ATP synthase activity, increased membrane potential, decreased oxygen consumption, and increased ROS with increasing IF1 dose. Through transcriptomics and proteomics, the authors identified changes in the synaptic components as substantially affected by IF1 dose. The authors then examine postsynaptic response in the glutamatergic synapse between CA3 and CA1 neurons finding it increased with increased levels of IF1 and with a corresponding effect on memory. Finally, by using the antioxidant MitoQ the authors found that some of the memory benefits gained from higher levels of IF1 may be the result of IF1-driven ROS generation and ROS signaling. This is well-planned, well-written study that elegantly links subtle changes in respiration to synaptic function and behavior.*

*Minor points:*

*1- Human IF1 is used. The authors should comment on the conservation of IF1 between mouse and human and comment on any effect that human rather than mouse IF1 for overexpression may have.*

IF1 is a conserved protein in mammals and can inhibit the ATP synthase of different species, including yeast (Cabezon JBC, 2002, 227:41334-41341). Specifically, the sequence identity between the mature mouse and human IF1 proteins (81 residues) is very high (75.3%), showing 61 invariants and 10 additional conservative residues. Conservation is even higher in the N-terminal inhibitory domain (84%) that, as in the bovine counterpart (Gordon-Smith J Mol Biol 2001; 308:308-339), are also structurally disordered domains (Esparza-Moltó, CMLS 2017;74:2151-2166). Consistent with these observations, when we have expressed the human protein in different rodent and human cell lines and observed the inhibition of the activity of the ATP synthase and the concurrent metabolic reprograming of the cells to an enhanced glycolysis and production of mtROS (Sanchez-Cenizo JBC 2010; 285:25308-25313). Hence, we do not envision any relevant functional differences for the overexpression of human or mouse IF1. A comment in this regard has been incorporated in the revised version of the manuscript (Page 5).

*2- On page 5, the authors note that cristae are disorganized in the IF1 KO mice. They also note later that the ATP synthase oligomeric state is altered in the IF1 KO. Given, ATP synthase dimers affect cristae structure do the authors believe this to be a potential mechanism of the cristae abnormalities. The authors should comment on why cristae are observed to be disorganized in the IF1 KO.*

We thank the reviewer for his/her suggestion because IF1 might be a relevant protein for shaping cristae organization in some tissues by affecting the oligomerization state of the ATP synthase. In fact, IF1 co-fractionates with oligomeric assemblies of the enzyme (Esparza-Molto FASEB J 2019; Nuevo-Tapioles Nature Commun. 2020); its overexpression in vivo in mouse liver promotes the formation of ATP synthase dimers (Santacatterina, Oncotarget 2016); an increase in the mitochondrial dose of IF1 increases the oligomeric state of the enzyme (Supplemental Fig. S1) and it has been found bridging tetramers of ATP synthase in the cryo-EM structures of the complete enzyme (Gu Science, 2019; Pinke Nat Struct Mol Biol, 2020). This might explain why cristae are rather disorganized in mice lacking IF1 in neurons, while higher expression of IF1 and the concurrent inhibition of the ATP synthase results in more organized cristae architecture and efficient oxidative phosphorylation. A comment in this regard has been incorporated in the revised version of the manuscript (Page 16).

*3- The authors establish that IF1 is knocked out in the forebrain, but as this is a forebrain-specific conditional knockout, it needs to be shown that IF1 levels are unaffected in other tissues such as cerebellum or liver.*

In full agreement with the reviewer’s comment, a western blot showing the specific knockout of IF1 in forebrain when compared to cerebellum is now presented in the revised version of the manuscript (see new Figure S1A). As we reported previously (Esparza-Moltó, FASEB J, 2019), the content of IF1 in the heart and liver of various strains of mice is very low, almost negligible, when compared to that present in other mouse tissues such as brain, kidney or colon (see also new Figure S1A). Hence, no changes in the content of IF1 are observed in the liver or heart of mice upon the genetic ablation IF1 in forebrain neurons (see new Figure S1A). A comment in this regard has been incorporated in the revised version of the manuscript (Page 5).

*4- There is not a perfect correlation between the electrophysiology in 6A and the behavior in 6G- K, in that the KO has similar electrophysiology to the WT but a memory deficit at 2 hrs compared to WT. The authors should explicitly address reasons for this discrepancy in the discussion.*

We believe that the most likely explanation is that the activity of other synapses, either in the hippocampus (for example, dentate gyrus to CA3) or in other cortical regions, may be altered in IF1-KO mice and contribute to the impaired short-term memory of this genotype. Moreover, novel object recognition is a complex cognitive task, and it is unlikely to be fully explained by a single electrophysiological parameter. We now acknowledge this limitation in the Discussion section of the manuscript (Pages 17-18). We thank the reviewer for his/her observation in this regard.

*5- Fig. S2B shows decreased levels of NMDA receptor subunits in IF1 TG mouse samples. Does the gene expression data show decreased expression? If not, might this be mediated by receptor endocytosis and degradation due to increased stimulation by glutamate in IF1 TG mice (Fig. 5 shows increased glutamate levels in IF1 TG)? It would be helpful to state why NMDA receptor subunit levels are reduced in IF1 overexpression.*

Microarray data do not show a clear down-regulation of the expression of NMDA receptor subunits (Grin1, Grin2a, Grin2b) in IF1-TG mice, when compared to KO and CRL. Indeed, we confirmed this observation by qPCR for GluN1 (Grin1) and GluN2B (Grin2b) subunits (see new Figure S2C). Hence, the down-regulation of their expression in IF1-TG mice could result from their internalization and ubiquitin-dependent degradation, as suggested by the reviewer (see also Goo, Front Mol Neurosci, 2015, 8:60). Indeed, it is tempting to speculate that the reduction in the levels of NMDA receptors in IF1-TG mice might represent a mechanism to prevent neurotoxicity from excessive synaptic activity. In this regard, it has been described that prolonged increases in neuronal activity *in vitro* by the treatment with bicuculline, an antagonist of the inhibitory GABA_A_ receptors, promotes the ubiquitination and down-regulation of GluN1 mediated by the E3 ubiquitin ligase Fbx2 (Kato et al., PNAS, 2005, 102:5600-5605). In line with this argument, the increased neuronal activity in TG mice may be due to the greater phosphorylation of AMPA receptors and the enlargement of dendritic spines. A comment in this regard has been incorporated in the revised version of the manuscript (Page 11).

**Reviewer #2:**

*In this article by Esparza-Moltó et al., the authors have investigated the effects of overexpressing and knocking out the protein IF1, an inhibitor of the ATP synthase. They have taken multi-pronged approaches to characterize the impact of IF1 knockout and overexpression on mitochondrial structure, function, neuronal transcriptomics, proteomics, metabolomics, electrophysiology, and cognitive tasks. I recommend this paper for publishing provided the following questions are addressed:*

*Figure 1H: at higher IF1 levels, the mitochondria structures are elongated, and in the knockout condition, they are rounder. Is that fragmentation? If yes, isn't mitochondria fragmentation an indicator of glycolytic enhancement for ATP synthesis? Could you please elaborate?*

We thank the reviewer for his/her observation on mitochondrial morphology. It is true that the expression of the pro-fission DRP1 protein is significantly diminished in IF1-TG mice when compared to IF1-KO and CRL mice (Figure 1I). This observation might support more mt-fragmentation and a more rounded shape of the organelle in the latter genotypes, despite expressing similar levels of OPA1, MFN2 as in IF1-TG mice (Figure 1I). However, we think that it is hard to state that mitochondria are more fragmented in IF1-KO mice because no differences in DRP1 expression between IF1-KO and CRL mice were found (Figure 1I), but the aspect ratio of mitochondria -which is an indication of mt-elongation- is significantly higher in CRL mice (Figure 1H). Moreover, calculation of the mitochondrial area is similar among the three genotypes (see new Figure 1H). Hence, we rather support that mitochondria are more rounded in IF1-KO mice because of their more disorganized cristae organization (see comment 2 of R#1). This situation compromises the efficiency of OXPHOS, as evidenced by the increased H^+^ leak in IF1-KO and CRL mice when compared to IF1-TG (Fig. 2B), and further correlates with the observed differences in glycolytic flux among the three genotypes (Figure 2E,F), to keep similar ATP/ADP ratios (Figure 2H). A comment in this regard has been incorporated in the revised version of the manuscript (Pages 5 and 16).

*2. Figure 1E: the experiment should be performed in the presence of a glycolysis inhibitor + mitochondrial substrates (pyruvate or lactate) to show that the observed ATP synthesis increase is due to an increase in mitochondrial ATP production and not glycolysis.*

There is some misunderstanding here. In Figure 1E we determined the rates of ATP production in isolated brain mitochondria using as respiratory substrate succinate, a Complex II electron donor. We should mention that our mitochondrial preparations are essentially devoid of glycolytic contaminants. Moreover, we included the corresponding control experiments using oligomycin, which is an inhibitor of the ATP synthase, to show that the reduction in the rates of mitochondrial ATP synthesis resulted from the inhibition of the enzyme as the dose of IF1 increased. This point is more clearly presented in the revised version of the manuscript (see new Figure 1 legend in Page 45).

*3. Could you please explain how IF1 increase reduces glycolytic flux? For instance, are glucose transporters reduced on the membrane surface that results in lower glucose intake?*

In addition to the regulation of glycolytic enzymes by the energy charge and redox state of the cells, which are directly influenced by the efficiency of OXPHOS, and as we have already mentioned under point 1, we support that the increase in IF1 dose might contribute to reduce the glycolytic flux of neurons by better organizing mitochondrial cristae. Functionally, by promoting a more efficient respiration with less dissipation of the proton gradient generated by the respiratory chain through the H^+^ leak, which we have found that inversely correlates with the mitochondrial dose of IF1 (Figure 2B). Additionally, the possibility exists that IF1 dose could affect the abundance of glucose transporters on the membrane. However, we have not investigated this possibility because none of the omic analyses suggested an alteration in glucose metabolism. A comment in this regard has been incorporated in the revised version of the manuscript (Page 17).

*4. Figures 3 and 4: the proteomics and transcriptomics analyses results denote downregulation of LTP in IF1 overexpressed neurons. However, the electrophysiology measurements and the animal behavior experiments show otherwise. Please explain.*

The downregulation of the LTP IPA category is dominated by the reduction in the expression of genes such as Mapk1, Map2k1 and Snca, and the observed reduction in NMDA receptor subunits (Fig. S2B). However, LTP itself is preserved in IF1-TG mice (Fig. S2A). As discussed above (see Reviewer #1, comment 5), the downregulation of NMDA receptors is likely a compensatory consequence for the hyperexcitability of the synaptic circuits in the IF1-TG mice (Fig. 6C), and it is also correlated with the reduction in the number of spines in these animals (Fig. 6E). The LTP category includes a complex network of cellular functions, and many proteins in this category have non-univocal relations with synaptic potentiation. For example, the transcript levels of Mapk1 (the gene encoding for ERK2) are reduced in IF1-TG mice when compared to KO (Fig. 3H). However, ERK2 phosphorylation (and therefore protein activation) is enhanced in the IF1-TG (Fig. 6D). Similarly, we observed an increase in the phosphorylation of AMPA receptors at residues associated with synaptic potentiation (pGluA1 S831, S845; Fig. 6D), even though LTP itself is not enhanced. In fact, these changes in IF1-TG mice are most in agreement with the enhanced synaptic transmission we observed in the electrophysiological study.

Regarding behavior, synaptic plasticity is considered the cellular basis for learning and memory and LTP at CA1 synapses is a prominent mechanism of plasticity. However, its direct involvement in memory is subject of debate (Zamanillo, Science, 1999, 284:1805-1811; Bannerman, Nat Neurosci, 2012, 15:1153-1159; Valbuena, Nat Commun, 2019, 10:4991). In our study, enhanced long-term memory in IF1-TG mice is better correlated with increased basal transmission and ERK signaling rather than with LTP expression at CA1 synapses. In this case, we suggest that IF1 inhibition of the ATP synthase contributes to improved cognitive function, perhaps because of the enhanced neuronal activation triggered by ERK signaling. A comment in this regard has been incorporated in the revised version of the manuscript (Pages 17-18).

*5. The resolution of the figures needs to be improved. Some of the font sizes also need to be made more prominent.*

We have checked the resolution and font sizes of the Figures and detected no problem when compared to PLOS Biology requests.

**Reviewer #3:**

*In this overall well-executed study, the authors used newly generated genetic strains of ATPase inhibitory factor 1 (IF1) to characterize the effects of loss- and gain-of-function of IF1 in the mouse. Results clearly showed that IF1 interacts with ATPase under physiological condition to define the fraction of active/inactive enzyme in vivo, and that IF1 dose critically regulates mitochondrial metabolism, synaptic function, and learning and memory. Moreover, the main effect of IF1 appears to be mediated by mitochondrial ROS, as the treatment of IF1 Tg mice with the mitochondrial-targeted antioxidant mitoQ effectively blocked the synaptic and cognitive effects of IF1 overexpression in the Tg animals. Overall, the experiments are well executed with sufficient statistical analysis, the conclusion is well supported, and the take home message is clear: Regulation of mitochondrial ROS signaling by IF1 is important for synaptic function and cognitive behavior in mammals.*

*One point that is missing from the study is the mechanism of mitochondrial ROS regulation by IF1. Since mitochondrial complex I is a major source of mito-ROS, and one way for IF1 to affect ROS production by complex I is through reverse electron transfer (RET). It would add more mechanistic insight to the study if the authors can test the involvement of RET in IF1-induced mito-ROS production using RET inhibitors described in the literature.*

In agreement with the reviewer’s comment, we have studied reverse electron transfer (RET) at Complex I because it is a relevant signaling pathway operating *in vivo* during ischemia (Chouchani et al., Nature 2014; 515:431-435; Cell Metab 2016;23,254-263), in inflammation (Mills et al., Cell 2016; 167: 457-470), life span extension and as part of the oxygen-sensing mechanism of the carotid body (Scialo et al., Cell Metab. 2016; 23:725-734). In this regard, RET was determined in isolated brain mitochondria of IF1-KO, CRL and IF1-TG mice with the Amplex Red oxidation protocol using succinate as respiratory substrate in the absence or presence of rotenone or FCCP (Robb, JBC 2018, 293:9869–9879; Yin, Nat Commun 2021, 12:707). Interestingly, we observed that isolated brain mitochondria have a substantial rate of H_2_O_2_ generation sensitive to rotenone. However, there are no significant differences in basal RET (rotenone-sensitive H_2_O_2_ production rate) among the three genotypes (S1E Fig). Anyway, since RET levels *in vivo* are controlled by ΔΨm (Robb, JBC 2018), our findings support that RET enhances its contribution to mtROS production as the dose of IF1 increases ΔΨm (Figure 2I). These results have been incorporated in the revised version of the manuscript (Pages 7 and 15) and in new Figure S1E.

**Reviewer #4:**

*This is an ambitious paper that makes the case that ATPase inhibitory factor 1 (IF1), an inhibitor of mitochondrial ATP synthetase, regulates Esparza-Moltó synaptic function, learning and memory. Such a claim deserves close consideration and has bold implications because it amounts to saying that regulating ATP availability, a fundamental cellular metabolic function is sufficient to control neural circuit and network function. There are so many steps along the causal chain between ATP synthetase regulation and expression of memory, that it is head spinning to even contemplate, but the authors do a remarkable job of making a plausible case. The experiments are laid out like detectives aiming to meet the burden of proof of "a preponderance of the evidence" rather than a "beyond a reasonable doubt." In short, the work is compelling, but it leaves this reader with the uncomfortable conclusion that it is hard to understand how regulating ATP availability, tantamount to energy availability, leads to the cellular and behavioral manifestation of knowledge. Nonetheless, the evidence of a series of carefully selected and logical correlations is remarkable, and while the biochemistry and respiratory signaling is not my area of expertise, the data and implications are intriguing even if implausible.*

*I was especially impressed that the commercial antioxidant MitoQ, which like endogenous antioxidant CoQ10, reduces mitochondrial reactive oxygen species was able to block the effects of overexpression of IF1 in transgenic mice, arguing that quenching ROS, a result of ATP metabolism, is sufficient for regulating cellular functions like synaptic strength at the hippocampus Schaffer collaterals, and object recognition memory.*

We appreciate the reviewer’s observation that our data are intriguing and that we are making a plausible case for the link between ATP synthase regulation and cognitive function. Just to clarify, we do not believe that this link is established through ATP availability (in fact, ATP levels remain fairly constant in IF1-KO and TG animals; Fig. 2H). Our point is that the link is stablished via intracellular signaling triggered by the production of mitochondrial reactive oxygen species (mtROS) in response to IF1-mediated inhibition of the ATP synthase. This point is now more clearly presented in the revised version of the manuscript (see Pages 15-16) and supported by the obliteration of synaptic strength and object recognition memory when mtROS are quenched by MitoQ treatment, emphasizing the relevance of mtROS in synaptic function and cognition.

*While I confess, that I do not see any outright logical flaws, the conclusions are still unsettling and their generality questionable. Consequently, I'd like to see the same experiments done for another synapse and another behavior, perhaps a conditioned memory to understand how specific or general these effects are. Given that energy metabolism is so basic, one imagines the effects must generalize to many synapses and behaviors. I should point out that regardless of the result, the work deserves to be published, but it would be misleading as is, to publish work that implies such general effects without demonstrating the generality.*

About the specificity (or generality) of the effects we are describing here, we believe the core mechanisms are probably conserved across different cell types and brain regions, but the functional synaptic (and cognitive) outcomes may or may not generalize. For example, we have observed an enhanced ERK and CaMKIIα signaling in response to mtROS production in the hippocampus (Fig. 7D). This is probably behind the enhanced synaptic transmission at the CA3-CA1 synapse, which in turn may have led to a homeostatic downregulation of the number of dendritic spines and NMDA receptors, without altering LTP. The same initial signaling event may have triggered a different functional outcome at the dentate gyrus-CA3 synapse, which mostly relies on presynaptic, NMDA receptor-independent mechanisms for plasticity. On the other hand, fast-spiking interneurons (not affected in our Camk2a transgenic animals) are highly sensitive to metabolic and oxidative stress. In this case, enhancing mtROS signaling may overwhelm their capacity and produce deleterious effects for rhythm generation and information processing at cortical circuits. Therefore, we agree with the reviewer in that the generality or specificity of these mechanisms is an interesting conceptual issue (and it is now further addressed in the Discussion section, see pages 18 and 19), but we believe it would be outside the scope of this study to test it experimentally.

*The Schaffer collateral fEPSP waveforms are unusual. If the scale bars of 10 ms in Figs. 6 and 7 are correct then the responses are very slow. What is the Fig. 6B scale bar? It shouldn't take 10 ms for the fiber volley! Similarly, the fEPSP slopes seem to be off by an order of magnitude. More importantly, the waveshape of the fEPSP response are unusually complex in the transgenic and knockout mice. The "ringing" of population spikes suggest the slices are epileptic. That would make it very difficult to accurately measure the synaptic response, given ongoing seizure-like activity. The picrotoxin to block inhibition, likely has exacerbated this, but was unnecessary. In short, but consistent with the fundamental disruption that one expects by manipulating IF1, these mutant mice (or at least their hippocampal slices) do not appear to be normal in more fundamental features than the features that are measured by the stimulus response assessment.*

We apologize for not having explained ourselves properly at this point. Fig. 6B was drawn as a representative figure for illustration purposes to favor comprehension for non-specialists of a trace recorded in CA3-CA1 synapses. Therefore, it has no scale. We have also corrected the scale bars in Figs. 6C and 7C: the vertical axis depicts 0.2 mV.

As for the values of the fEPSP slopes presented in panels 6C and 7C, we agree with the reviewer in that they are rather small. They are not much smaller than other values published recently by the Esteban group (see for example Sánchez-Puelles et al, Cereb Cortex 2020 30:505-524), but they are definitively lower than average. We are not sure of the reason for these small postsynaptic responses. It may be related to the age of the animals used in this study (8-9 months) or some technical difference related to the stimulation efficiency. In any case, we are confident that we can compare absolute fEPSP values across genotypes (control, KO and TG with or without MitoQ), because all these recordings were carried out in parallel, in an interleaved manner.

Regarding the population spikes in the representative traces from IF1-TG mice (Fig. 6C and Fig. 7C), we should point out that these only appear at high stimulation intensities (at 150 μA or above; the representative traces at the insets correspond to 250 μA). Indeed, we did not observe ongoing epileptic activity in the absence of strong stimulation in the slices from the IF1-TG animals, and we could always measure the fEPSP slope from the beginning of the waveform, avoiding population spikes. Also, we have not detected seizures or any gross abnormality in the behavior of the IF1-TG animals, despite their increased basal excitatory synaptic transmission. Compensatory mechanisms may have also occurred at the level of inhibitory GABAergic transmission. We have not observed significant differences in the levels of total GABA in the hippocampus, but we cannot rule out that there are differences in the extracellular GABA or in the responsiveness of GABA receptors. In fact, our transcriptomic analysis indicated an upregulation of the GABRA2 gene (GABA-A receptor, alpha2 subunit) in TG animals.
